# Hypogenetic lung syndrome in an adolescent: Imaging findings with short review

**DOI:** 10.4103/1817-1737.39639

**Published:** 2008

**Authors:** Mohamed Firoze Ahamed, Fahad Al Hameed

**Affiliations:** *Department of Pulmonary, King Khalid National Guard Hospital, King Abdul Aziz Medical City, Jeddah, Saudi Arabia*

**Keywords:** Dextrocardia, pulmonary hypoplasia, sequestration

## Abstract

Hypogenetic lung syndrome is more popularly known as a scimitar syndrome (SS). It is a rare developmental lung malformation which almost always occurs on the right side. The two most constant features of this syndrome are anomalous pulmonary venous return into systemic circulation, most frequently via inferior vena cava (IVC), and lung hypoplasia.

We are reporting such a case illustrating most typical and some uncommon features on chest radiograph and multislice computer tomography (MSCT) of chest. Focal herniation of liver through a diaphragmatic defect presenting as an ovoid soft tissue mass in right lower paraspinal region on chest X ray mimicking sequestration is an interesting but rare finding.

With recent advances in postprocessing workstations, the various complex vascular and nonvascular anomalies in chest are very elegantly demonstrated in three-dimension (3-D) volume rendering and maximum intensity projection (MIP) reformatted images in multiple planes. These various 3-D reconstruction images give surgeons a clear understanding of the complex malformations and help in precise operative plans without conventional catheter angiographies.

## Case Report

A 15-year-old Saudi girl was referred to our respirology clinic with 2 years' history of slowly progressing and recurrent exertional dyspnea, chest pain, wheeze and fatigue. She denied any history of TB; smoking; exposure to chemicals, organic dust, toxic gases or bird feeds.

**Figure 1:**
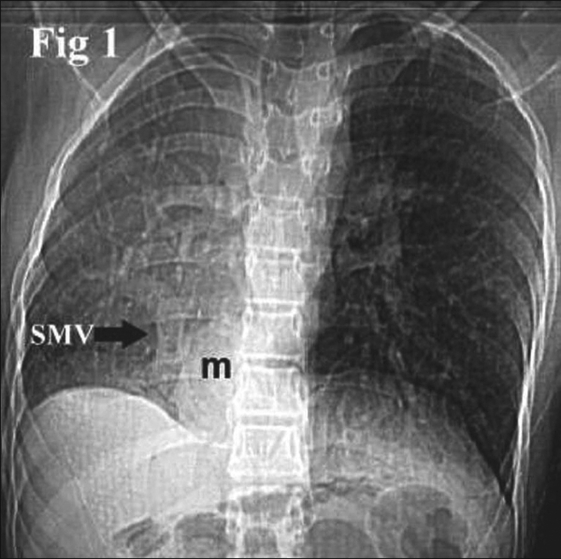
A frontal chest radiograph of a 15-year-old Saudi girl with scimitar syndrome, demonstrating a scimitar sign (msv), focal herniation of liver (m) mimicking a right paraspinal mass or sequestration, slightly opaque and small right hemithorax with nonvisualization of heart shadow

On physical examination, she was afebrile, dyspneic but not hypoxic. Her blood pressure was 115/65 mmHg, and her weight was less than the third percentile. Chest auscultation revealed wheeze and cardiac pulsations on the right side. Her initial chest radiograph [Figure 1] showed slightly small opaque right hemithorax with nonvisualization of heart, which is shifted to the same side; and a scimitar vein (scimitar sign). Also present was an ovoid soft tissue mass (m) of 3 × 4 cm size in right lower paraspinal region, medial to scimitar vein.

**Figure 2:**
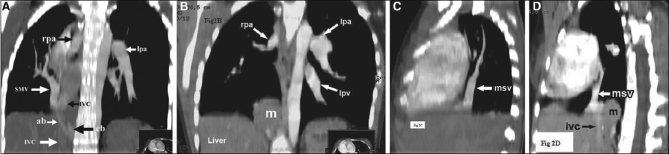
A-B, coronal; and C-D, sagittal reformatted MIP images demonstrating the main scimitar vein (msv) draining into inferior vena cava (IVC), hypoplastic right (rpa) and normal left (lpa) pulmonary arteries, focally herniated liver (m) mimicking mass and arterial branches from abdominal aorta (ab) and celiac trunk (cb) to right lung base

A diagnosis of scimitar syndrome (SS) with sequestration was considered and further imaged with multislice CT and echocardiography. The contrast-enhanced MSCT of chest confirmed the anomalous venous return from the hypoplastic right lung by a main scimitar vein (msv) into inferior vena cava just above the diaphragm [Figures 2A–D, 5]. What looked like a sequestration (m) on chest radiograph was, in fact, a part of the liver herniating through focal defect in the hemidiaphragm, well illustrated on reformatted MIP images [Figures 2B, 2D].

**Figure 3:**
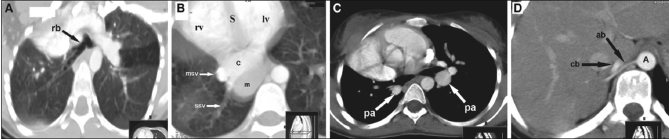
(A) An angled axial reformatted image on lung window at carinal level showing bilobed right bronchus (rb). (B) An axial scan at lung bases on lung window showing a small second anomalous vein (ssv) draining directly to IVC (c), just posterior to termination of larger scimitar vein (msv), and dextroposition of heart in small right hemithorax with anteriorly directed interventricular septum (s). An ovoid mass (m), posterior to IVC, is a part of herniated liver. Left ventricle (lv). Right ventricle (rv). (C) An axial image on mediastinal window, just below the carinal level demonstrating pulmonary arteries (pa), posterior to airways on both sides identically; left isomerism. Pulmonary artery on right side is hypoplastic. (D) A contrast-enhanced axial scan through upper abdomen on soft tissue window illustrating a small arterial branch (ab) from aorta (A) and a relatively bigger branch (cb) from celiac trunk. These branches could be followed to right lung base on serial images (not shown), confirming the systemic arterial supply to right lung base

**Figure 4:**
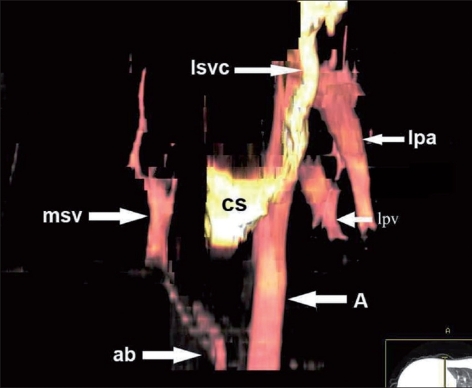
A 3-D colored volume rendering MIP image demonstrating main scimitar vein (msv), persistent left superior vena cava (lsvc) draining into coronary sinus (cs), systemic arterial branches (ab) from abdominal aorta and celiac trunk to right lung base. Left pulmonary artery (lpa), aorta (A). Left pulmonary vein (lpv)

Other associated abnormalities demonstrated on MSCT examination of our patient were:

- Hyparterial, bilobed right bronchus [Figure 3A]
- A second very small anomalous vein from right lung base draining directly into IVC posterior to the main scimitar vein [Figure 3B]
- Hypoplastic right main pulmonary artery and its branches [Figures 2B and 3C]
- Dextroposition of heart in small right hemithorax [Figure 3B]
- Systemic arterial supply to right lung base by two small branches [arrows in Figure 3D and Figures 4A, 4B] coming from celiac axis and lower thoracic aorta (A)
- A persistent left superior vena cava, draining to coronary sinus [Figure 4]

IVC below the diaphragm was intact, and no pulmonary veins draining to left atrium were identified on the right side.

An echocardiograhic examination was normal.

## Discussion

Hypogenetic lung syndrome, also known as congenital venolobar syndrome or scimitar syndrome (SS), is primarily a complex developmental lung abnormality with anomalous venous return. The most common features are lung hypoplasia, anomalous pulmonary venous return to IVC, pulmonary artery hypoplasia, bronchial anomalies and systemic arterial supply to hypoplastic lung. It almost always occurs on the right side, slightly more common in women (1.4:1); but there are rare cases reported on the left side and very rarely bilateral.[1] It involves hypoplasia of one or more lobes, commonly upper and midlobes of the right lung. Occasionally, the affected lung may be monolobed with no fissures or bilobed without horizontal fissure and very rarely of horseshoe type. The right bronchus is usually bilobed and hyparterial, similar in morphology to left lung, i.e., left isomerism or bilateral left lungs. These lung anomalies are important in distinguishing scimitar syndrome from simple lung hypoplasia and isolated partial anomalous pulmonary venous return. The hypoplastic but not sequestrated lung may also receive systemic blood supply - at least to a part, most frequently the posterior basal segment directly from aorta or celiac axis. These arterial branches generally enter the lung through inferior pulmonary ligament and are believed to represent persistent embryonic aortic postbranchial arches supplying lung buds before the development of main pulmonary artery. The pulmonary artery hypoplasia determines the degree of systemic arterial supply to hypoplastic lung. In most patients, pulmonary artery hypoplasia is mild. However, it may be very significant or even absent.[2–4] In our reported case, the right pulmonary artery was hypoplastic and there was systemic supply to right lung base by two small branches, one from celiac axis and the other directly from abdominal aorta [Figure 3D].

The other most constant component of this syndrome is an anomalous pulmonary vein or veins draining at least a part or the entire affected lung most commonly to inferior vena cava just above or below the diaphragm. Uncommonly, the anomalous vein may drain into hepatic, portal, azygos veins; coronary sinus; or right atrium. This results in extracardiac left-to-right shunt, which is usually mild in most patients and determines the clinical presentation and symptomatology. If the shunt ratio is more than 2:1, there is increased risk of pulmonary hypertension and right heart failure over a period of time.[5]

About a quarter of the affected individuals have associated sinus venosus type of atrial septal defect. Ventral septal defect, patent ductus arteriosus, aortic coarctation, tetralogy of Fallot, double-chamber right atrium, hypoplastic left heart and endocardial cushion defects are the other rare associations.[2,5] Mediastinal shift and cardiac dextroposition are frequent findings, which largely depend on degree of lung hypoplasia. The right heart border or the entire heart may be indistinct, as in this case; and in some patients, the scimitar vein may be obscured by dextroposition of the heart on chest radiographs. Pulmonary sequestration, commonly of extralobar type, is seen in about a quarter of patients. Persistent left superior vena cava and hemidiaphragm anomalies like eventration, focal defect, accessory diaphragm and Bockdalek hernia may also occur. Genitourinary and spine anomalies, phrenic cyst and absence of left pericardium are some other very rare associations of this syndrome.[2,4,6]

In 1836, Raul Chassinat of France and George Cooper of UK first described this syndrome independently on autopsy but did not compare the anomalous pulmonary vein to a scimitar. A century later, in 1956, Halasz NA *et al.* reported a similar case and compared the anomalous pulmonary vein to a scimitar. The term ‘scimitar syndrome,’ however, was first coined by Neill *et al.* in 1960; and Felson B in 1973 called it ‘congenital pulmonary venolobar syndrome.’ The literal meaning of *scimitar* is ‘a short curved sword with an edge on the convex side, used chiefly by Turks and Arabs’ (Webster's dictionary). A scimitar vein is a vertical curvilinear opacity in right mid-lower lung, running along the right heart border inferomedially towards diaphragm to join IVC. A scimitar vein when present on a frontal chest radiograph is called ‘scimitar sign,’ which was first coined by Dother *et al.* in 1949. Scimitar syndrome was described in literature by many other interesting names like Halasz syndrome, anomalous pulmonary venous return/connection, Turkish sabre syndrome, epibronchial right pulmonary artery syndrome, Signo de La cinitarra (Italy), vena cava bronchovascular syndrome, Krumsubel syndrome (Germany), etc.[7,8]

Scimitar sign and scimitar syndrome are not synonymous. An isolated partial anomalous pulmonary venous return may be present with scimitar sign. Rarely, a scimitar vein courses down towards the medial costophrenic angle to drain into IVC and left atrium and is called scimitar variant. A wandering or meandering pulmonary vein resembling a scimitar on frontal chest X rays is reported to drain into left atrium without any connection to IVC. This is called by some authors as pseudoscimitar syndrome and may be associated with lung hypoplasia and systemic arterial supply.[9–11]

Although the etiology of scimitar syndrome is unknown, a majority of these are sporadic; but some are familial and transmitted as an autosomal dominant trait. Frequency is about 1-3 per million live births or even more as majority of asymptomatic patients remain undiagnosed.

Three forms of scimitar syndrome have been described in the literature: (i) infantile, (ii) adolescent and (iii) adult:[12]

- Patients presenting in infancy have more severe symptoms, higher incidence of pulmonary hypertension and heart failure. Majority of them have cardiac anomalies, most commonly ASD and VSD; and the left-to-right shunt resulting from scimitar vein is considerably large. An obstructed scimitar vein and systemic supply to hypoplastic lung further contribute to severity of pulmonary hypertension. The pulmonary overcirculation can prevent the normal postnatal regression of pulmonary artery muscularity, resulting in persistent pulmonary hypertension of newborn.[13,14]
- In the adolescent type, the clinical symptomatology mainly depends on the degree of lung hypoplasia and presents commonly with wheeze, shortness of breath, fatigue and failure to thrive, like in our case.
- In adults, the scimitar syndrome is diagnosed incidentally on chest radiographs obtained for other reasons, as most are asymptomatic. They typically have no associated cardiac anomalies, and left-to-right shunt is insignificant.

## Conclusion

Scimitar syndrome is a rare complex congenital abnormality of the chest, frequently diagnosed on chest radiographs by presence of the scimitar vein. At times this vein may be obscured by extreme cardiac dextroposition or may be very thin, resulting in misdiagnosis of lung hypoplasia, dextrocardia or Sawyer-James syndrome. Rarely, a meandering pulmonary vein, scimitar variants and pseudoscimitar syndrome are easily mistaken for scimitar syndrome on chest X rays. Therefore, these individuals must be further evaluated with MSCT or MR angiography. Multislice CT with multiplanar and three-dimensional image reconstruction not only elegantly demonstrates the anatomy of various vascular anomalies but also gives a detailed account of common and rare known associations. This also helps in precise planning of the surgery and obviates more invasive procedures like cardiac catheterization and conventional angiography.

Focal herniation of liver through a defect in diaphragm is reported rarely,[15] and it presenting like a sequestration or mass on chest radiograph makes this case report interesting.
